# Spectral properties and the accuracy of mean-field approaches for epidemics on correlated power-law networks

**DOI:** 10.1103/PhysRevResearch.1.033024

**Published:** 2019-10-15

**Authors:** Diogo H. Silva, Silvio C. Ferreira, Wesley Cota, Romualdo Pastor-Satorras, Claudio Castellano

**Affiliations:** ^1^ Departamento de Física, Universidade Federal de Viçosa, 36570-900 Viçosa, Minas Gerais, Brazil; ^2^ National Institute of Science and Technology for Complex Systems, 22290-180 Rio de Janeiro, Rio de Janeiro, Brazil; ^3^ Departament de Física, Universitat Politècnica de Catalunya, Campus Nord B4, 08034 Barcelona, Spain; ^4^ Istituto dei Sistemi Complessi (ISC-CNR), Consiglio Nazionale delle Ricerche, Via dei Taurini 19, 00185 Roma, Italy

## Abstract

We present a comparison between stochastic simulations and mean-field theories for the epidemic threshold of the susceptible-infected-susceptible model on correlated networks (both assortative and disassortative) with a power-law degree distribution $P\left(k\right)∼k^{-\gamma }$. We confirm the vanishing of the threshold regardless of the correlation pattern and the degree exponent γ. Thresholds determined numerically are compared with quenched mean-field (QMF) and pair quenched mean-field (PQMF) theories. Correlations do not change the overall picture: The QMF and PQMF theories provide estimates that are asymptotically correct for large sizes for γ<5/2, while they only capture the vanishing of the threshold for γ>5/2, failing to reproduce quantitatively how this occurs. For a given size, PQMF theory is more accurate. We relate the variations in the accuracy of QMF and PQMF predictions with changes in the spectral properties (spectral gap and localization) of standard and modified adjacency matrices, which rule the epidemic prevalence near the transition point, depending on the theoretical framework. We also show that, for γ<5/2, while QMF theory provides an estimate of the epidemic threshold that is asymptotically exact, it fails to reproduce the singularity of the prevalence around the transition.

## I. INTRODUCTION

Metabolic chains of protein interactions [1], collaborations among scientists, co-starring in a movie [2], and person-to-person contacts [3] are all examples of interacting systems that can be modeled using complex networks [2]. A large number of networks representing real systems show a heavy-tailed degree distribution described by a power law $P\left(k\right)∼k^{-\gamma }$ [4,5], usually with strong levels of correlations [6,7]. Degree correlations are encoded in the conditional probability $P(k^{\prime }|k)$ that a vertex of degree k is connected to a vertex of degree $k^{\prime }$ [6]. Technological networks, such as the Internet, show in general disassortative mixing [6,7], i.e., vertices of large degree tend to be connected with those of small degree and vice versa. Assortative mixing occurs in social networks, where connections preferentially occur among vertices exhibiting similar degree. Since uncorrelated networks usually simplify theoretical approaches, they are typical benchmarks for the investigation of dynamical processes on networks and have been considered in many studies [8–10]. However, the ubiquitousness of correlations in real networks naturally calls for the investigation of the effect of correlated interaction patterns. While the effects of degree correlations have been considered for several dynamical processes [11–16], a full understanding of their effects on the performance of theoretical approaches is still missing.

A basic approach to investigate dynamical processes on networks is the heterogeneous mean-field (HMF) theory, in which degree heterogeneity and correlations are taken into account through the distributions P(k) and $P(k|k^{\prime })$, respectively [8,9,17,18]. A more refined approach is provided by the quenched mean-field (QMF) theory [19–21], which considers the full topology as described by the unweighted adjacency matrix (defined as $A_{ij}=1$ if vertices i and j are connected and $A_{ij}=0$ otherwise) and thus takes into account the detailed connectivity structure.

A crucial question in this context is the ability of theories to accurately predict the epidemic threshold of the susceptible-infected-susceptible (SIS) dynamics, the most basic epidemic process with an absorbing-state phase transition [21–28]. For random uncorrelated networks, such as those created according to the uncorrelated configuration model [29], when γ<5/2 the two theories tend to agree, predicting a vanishing threshold as the network size diverges [22]. For γ>3 instead, QMF theory correctly predicts again the asymptotic vanishing of the epidemic threshold [30], while the HMF theory fails, predicting the existence of a finite threshold. In spite of being qualitatively correct, QMF theory is however not able to accurately predict the effective finite-size epidemic threshold in this case [25]. A further quantitative improvement of the QMF theory has been achieved in Ref. [26] [hereafter called of pair QMF (PQMF) theory] by means of the explicit inclusion of pairwise dynamical correlations [31–33]. See [34] for a recent application of the PQMF theory in epidemic containment.

In this work we investigate the ability of the aforementioned approaches (HMF, QMF, and PQMF) to quantitatively predict the value of the epidemic threshold for both uncorrelated and correlated networks generated using the Weber-Porto model [35] and for real-world topologies. We find that correlations do not change qualitatively the scenario found on uncorrelated networks. The epidemic threshold vanishes asymptotically with the system size for both assortative and disassortative correlations. For γ<5/2, both QMF and PQMF theories seem to provide an asymptotically exact estimate of the numerical threshold, while they are only qualitatively correct for γ>5/2. As in the case of uncorrelated networks [26], PQMF theory outperforms the other theories. The amplitude of the discrepancies between numerics and theory is correlated with violations of the assumptions underlying them, revealing that both theories tend to be more accurate if the principal eigenvector of the (effective) adjacency matrix is not strongly localized or the spectral gap is large. The same scenario is found to hold when SIS dynamics is considered on a set of real-world topologies. In addition, we analyze the singularity of the prevalence near the transition point through the critical exponent β, defined as $\rho ∼{\left(\lambda -\lambda_{c}\right)}^{\beta }$. Interestingly, we find that for γ<5/2, even if the QMF theory provides an asymptotically exact estimate of the position of the epidemic threshold, the QMF prediction for the prevalence exponent, $\beta^{\text{(QMF)}}=1$ [23,36], is correct only not too close to the transition.

The rest of the paper is organized as follows. Section II describes the models used to generate correlated heavy-tailed networks, the implementation of the SIS model, and the theoretical approaches. A comparison between simulations and theory on synthetic and real networks in presented in Sec. III. A summary is given and conclusions are drawn in Sec. IV. The Appendix summarizes the properties of the real networks investigated.

## II. MODELS AND METHODS

### A. Weber-Porto configuration model

The degree correlations encoded in the conditional probability $P(k^{\prime }|k)$ can be more easily interpreted by the simple metrics of the average degree of the nearest neighbors as a function of the vertex degree [6], defined as

(1) $$\kappa_{\text{nn}}\left(k\right)=\sum_{k^{\prime }=k_{\text{min}}}^{k_{\text{max}}}k^{\prime }P\left(k^{\prime }|k\right),$$

where $k_{\text{min}}$ and $k_{\text{max}}$ are the lower and upper cutoffs of the degree distribution. If $\kappa_{\text{nn}}\left(k\right)$ increases or decreases with k, the networks are assortative or disassortative, respectively. In the case of uncorrelated networks we have [6]

(2) $$P\left(k^{\prime }|k\right)=P_{\text{e}}\left(k^{\prime }\right)=k^{\prime }P\left(k^{\prime }\right)/\langle k\rangle ,$$

which implies that $\kappa_{\text{nn}}=\langle k^{2}\rangle /\langle k\rangle ={\langle k\rangle }_{\text{e}}$ does not depend on k. We use here the edge distribution average ${\langle A(k)\rangle }_{\text{e}}=\sum_{k}A\left(k\right)P_{\text{e}}\left(k\right)$, where $P_{\text{e}}\left(k\right)$ is the probability that an edge ends on a vertex of degree k.

We are interested in heavy-tailed networks with degree distribution $P\left(k\right)∼k^{-\gamma }$ and correlation given by $\kappa_{\text{nn}}\left(k\right)∼k^{\alpha }$. These networks can be generated using an algorithm proposed by Weber and Porto [35], hereafter called the Weber-Porto configuration model (WPCM). The degree of each vertex is drawn according to the degree distribution P(k) and initially each node has k unconnected stubs. Two stubs are randomly chosen and connected with probability

(3) $$P_{\text{link}}\left(q^{\prime },q\right)=\frac{f(q^{\prime },q)}{f_{\text{max}}},$$

where q and $q^{\prime }$ are the respective degrees of the chosen vertices and $f_{\text{max}}$ is the maximum value of

(4) $$f\left(q,q^{\prime }\right)=1+\frac{\left[\kappa_{\text{nn}}\left(q\right)-{\langle k\rangle }_{\text{e}}\right]\left[\kappa_{\text{nn}}\left(q^{\prime }\right)-{\langle k\rangle }_{\text{e}}\right]}{{\langle k\kappa_{\text{nn}}\rangle }_{\text{e}}-{\langle k\rangle }_{\text{e}}^{2}},$$

computed over the whole network. Self- and multiple connections are forbidden. In the absence of degree correlations, we have $\kappa_{\text{nn}}={\langle k\rangle }_{\text{e}}$, implying $f(q,q^{\prime })=1$ and $P_{\text{link}}=1$. See Ref. [35] for more details.

**FIG. 1.**
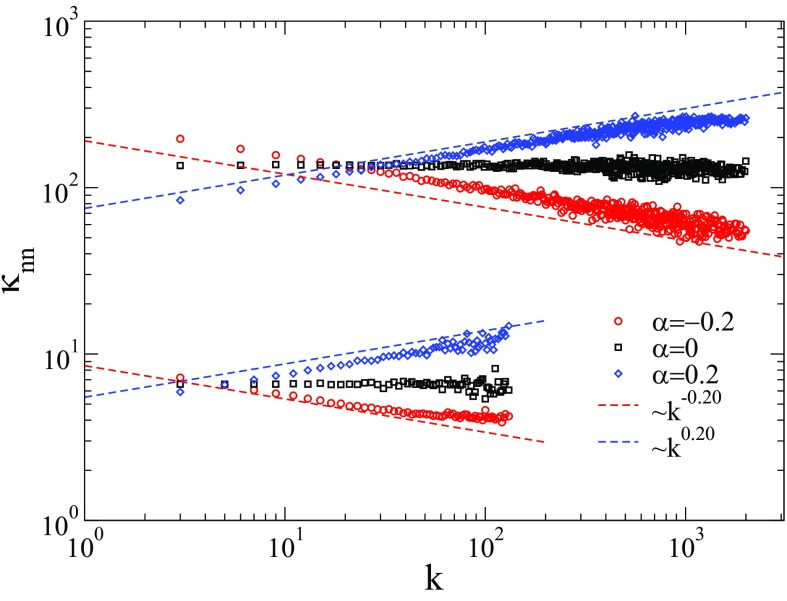
Average degree of the nearest neighbors as a function of the degree for networks built with the WPCM algorithm [35] for power-law degree distributions with γ=2.3 (top curves) and γ=3.5 (bottom curves). The network size is $N=10^{6}$ and the lower cutoff is $k_{\text{min}}=3$. The upper cutoff is given by $k_{\text{max}}=2\sqrt{N}$ for γ=2.3 and $NP(k_{\text{max}})=1$ for γ=3.5.

Figure 1 shows $\kappa_{\text{nn}}$ as a function of k for networks obtained with the WPCM algorithm [35] using different values of γ and α, with lowest degree $k_{\text{min}}=3$. We adopt different upper cutoffs for the degree distribution. For γ<3, the structural cutoff $k_{\text{max}}=2\sqrt{N}$ [37] is used, while for γ>3, a rigid cutoff is determined by the condition $NP(k_{\text{max}})=1$ [38]. The first choice allows us to enhance the effects of hubs and to approach faster the thermodynamic limit while fulfilling the criterion $k_{\text{max}}<\sqrt{\langle k\rangle N}$ necessary to produce uncorrelated networks in the case α=0 [37]. The second choice is justified by numerical reasons explained in Sec. II B. The predetermined scaling law $\kappa_{\text{nn}}\left(k\right)∼k^{\alpha }$ is very well reproduced. Small deviations for positive or negative α are due to the network finite size that prevents $\kappa_{\text{nn}}$ from decaying or increasing indefinitely with k. The range of the power-law behavior is extended as the network size increases.

### B. SIS simulations

In the SIS model, each edge of an infected vertex transmits the epidemic with rate λ, while infected nodes recover spontaneously with constant rate μ. The latter is fixed to μ=1 without loss of generality. The model can be simulated with the optimized Gillespie scheme proposed in Ref. [22]. See also Ref. [39] for more details.

We consider quasistationary simulations [40] in which the dynamics returns to a previously visited active configuration whenever the absorbing state, consisting of all vertices susceptible, is visited. This strategy permits us to circumvent the difficulties of dealing with the absorbing state, which is the only true stationary state for any finite-size networks. More details can be found in Refs. [39,41].

The effective transition point $\lambda_{c}\left(N\right)$, above which the epidemic remains in an active phase for very long periods, can be estimated using the position of the maximum of the dynamical susceptibility [22]

(5) $$\psi =N\frac{\langle \rho^{2}\rangle -{\langle \rho \rangle }^{2}}{\langle \rho \rangle }.$$

The choice of structural (for γ<3) and rigid (for γ>3) upper cutoffs allows the determination of the epidemic threshold unambiguously, avoiding multiple peaks and the smearing of the transition that can appear for SIS on power-law degree distribution networks, especially with large values of γ [22,27].

### C. Mean-field theories for correlated networks

In this section we summarize the predictions of the theoretical approaches that will be compared to numerical simulations in Sec. III. For QMF and PQMF approaches, the equations for uncorrelated and correlated networks are formally the same: Correlations have only the effect of modifying the entries of the adjacency matrix $A_{ij}$.

#### 1. Correlated heterogeneous mean-field theory

Heterogeneous mean-field theory takes into account nearest-neighbor correlations by the explicit consideration of the conditional probability $P(k^{\prime }|k)$. The HMF equation for the density of infected vertices with degree $k,\;\rho_{k}$, is given by [42]

(6) $$\frac{d\rho_{k}}{dt}=-\rho_{k}+\left(1-\rho_{k}\right)\lambda \sum_{l}kP\left(l|k\right)\rho_{l},$$

which yields an epidemic threshold given by

(7) $$\lambda_{c}\Upsilon^{\left(1\right)}=1,$$

where $\Upsilon^{\left(1\right)}$ is the largest eigenvalue of the connectivity matrix $C_{kl}=kP\left(l|k\right)$. For WPCM networks we have $P\left(l|k\right)=P_{\text{e}}\left(l\right)f\left(l,k\right)$, and therefore

(8) $$C_{kl}=\frac{klP(l)}{\langle k\rangle }f\left(l,k\right).$$

In the absence of correlations, $C_{kl}=\frac{klP(l)}{\langle k\rangle }$, implying that $\lambda_{c}=\frac{\langle k\rangle }{\langle k^{2}\rangle }$ [11,42]. It has been shown [11] that the HMF threshold vanishes for scale-free networks with 2<γ<3 in the thermodynamic limit, irrespective of degree correlations.

#### 2. Quenched mean-field theory

According to the QMF theory, which neglects pairwise dynamical correlations, the evolution of the probability $\rho_{i}$ that a vertex i is infected is given by [20]

(9) $$\frac{d\rho_{i}}{dt}=-\rho_{i}+\lambda \left(1-\rho_{i}\right)\sum_{j=1}^{N}A_{ij}\rho_{j},$$

where N is the network size. The epidemic threshold is given by

(10) $$\lambda_{\text{c}}^{\text{QMF}}\Lambda^{\left(1\right)}=1,$$

where $\Lambda^{\left(1\right)}$ is the largest eigenvalue (LEV) of the adjacency matrix $A_{ij}$. In the steady state we have

(11) $$\rho_{i}=\frac{\lambda \sum_{j}A_{ij}\rho_{j}}{1+\lambda \sum_{j}A_{ij}\rho_{j}}.$$

Using Eq. (11), Goltsev *et al.* [23] have shown that $\rho_{i}∼v_{i}^{\left(1\right)}$ for $\lambda ≳\lambda_{c}^{\text{QMF}}$, where $\left\{v_{i}^{\left(1\right)}\right\}$ is the principal eigenvector (PEV) corresponding to the LEV of $A_{ij},\;\sum_{i}A_{ij}v_{j}^{\left(1\right)}=\Lambda^{\left(1\right)}v_{i}^{\left(1\right)}$. Thus, the order parameter $\rho =\sum_{i}\rho_{i}/N$ of the QMF theory vanishes at $\lambda_{c}^{\text{QMF}}$ as

(12) $$\rho ≃a_{1}\left(\lambda \Lambda^{\left(1\right)}-1\right),$$

where

(13) $$a_{1}\left(N\right)=\frac{\sum_{i=1}^{N}v_{i}^{\left(1\right)}}{N\sum_{i=1}^{N}{\left[v_{i}^{\left(1\right)}\right]}^{3}}.$$

This same result was obtained independently in Ref. [36].

Within the QMF framework, Eq. (12) works well, close to the threshold $\lambda_{c}^{\text{QMF}}$, under the hypothesis that the network presents a spectral gap, i.e., the second largest eigenvalue of $A_{ij}$ is much smaller than the first, $\Lambda^{\left(1\right)}\gg \Lambda^{\left(2\right)}$. According to Eqs. (12) and (13), the QMF theory predicts the existence of an endemic state, with a finite fraction of infected vertices above the threshold $\lambda_{c}^{\text{QMF}}=1/\Lambda^{\left(1\right)}$, only if $a_{1}∼O\left(1\right)$, which occurs when the PEV is delocalized. Localization can be quantified by the inverse participation ratio (IPR) for the normalized PEV [23], defined as

(14) $$Y_{4}=\sum_{i=1}^{N}{\left[v_{i}^{\left(1\right)}\right]}^{4}.$$

If the PEV is delocalized then $Y_{4}∼N^{-1}$, while $Y_{4}∼O\left(1\right)$ if the PEV is localized on a finite number of vertices, but weaker forms of localization can be observed [43].

For random uncorrelated power-law networks the PEV is always localized [43]. For γ<5/2 it is (weakly) localized on a subextensive set of nodes coinciding with the maximum K-core, a subgraph of strongly mutually interconnected nodes with degree larger than or equal to K [44]. In such a case $Y_{4}∼N^{(\gamma -3)/2}$. For γ>5/2 it is instead strongly localized on the largest hub plus its nearest neighbors and $Y_{4}∼O\left(1\right)$ [45]. Hence, within QMF theory the threshold separates the absorbing phase from an active but strictly nonendemic state. However this does not imply that QMF predictions are necessarily flawed. Equation (9) factorizes the state of nearest neighbors and thus neglects dynamical correlations among them. These dynamical correlations actually transmit the infection from the localized PEV to the rest of the network and thus may in principle transform the active but localized state just above $\lambda_{\text{c}}^{\text{QMF}}$ into a full-fledged endemic state [25,46].

#### 3. Pair quenched mean-field theory

An improvement with respect to QMF theory is obtained by taking into account some dynamical correlations using the pairwise approximation developed in Ref. [26], where all derivation details can be found. Consider the probability $\phi_{ij}$ that a vertex i is susceptible and a neighbor j is infected. The dynamical system to be solved is

(15) $$\frac{d\rho_{i}}{dt}=-\rho_{i}+\lambda \sum_{j}\phi_{ij}A_{ij}$$

and

(16) $$\begin{array}{ccc} \frac{d\phi_{ij}}{dt} & = & -\left(2+\lambda \right)\phi_{ij}+\rho_{j}+\lambda \sum_{l}\frac{\omega_{ij}\phi_{jl}}{1-\rho_{j}}\left(A_{jl}-\delta_{il}\right)\\ & & -\lambda \sum_{l}\frac{\phi_{ij}\phi_{il}}{1-\rho_{i}}\left(A_{il}-\delta_{lj}\right), \end{array}$$

where $\omega_{ij}=1-\phi_{ij}-\rho_{i}$.

Here we develop a bit further the theory to analyze the steady state near the critical point. Keeping only leading terms up to second order in $\rho_{i}$ in Eq. (16), we obtain

(17) $$\phi_{ij}\approx \frac{\left(2+\lambda \right)\rho_{j}-\lambda \rho_{i}}{2+2\lambda }-\rho_{i}\rho_{j}+O\left(\rho^{3},\lambda \rho^{2}\right),$$

where we kept only leading order in $\lambda \approx \lambda_{c}\ll 1$ [26] for quadratic terms in $\rho_{i}$. Plugging Eq. (17) in Eq. (15) with $d\rho_{i}/dt=0$, we obtain

(18) $$\rho_{i}=\frac{\lambda \sum_{i}B_{ij}\left(\lambda \right)\rho_{j}}{1+\lambda \sum_{i}B_{ij}\left(\lambda \right)\rho_{j}},$$

where

(19) $$B_{ij}=\frac{2+\lambda }{2\lambda +2}\frac{A_{ij}}{1+\frac{\lambda^{2}k_{i}}{2\lambda +2}}≃\frac{A_{ij}}{1+\frac{\lambda^{2}k_{i}}{2}}$$

is an effective weighted adjacency matrix. The last passage in Eq. (19) assumes λ≪1.

Equation (18) has exactly the same form of the stationary $\rho_{i}$ in Eq. (11), obtained for QMF theory, replacing $A_{ij}$ by $B_{ij}$. Therefore, all the spectral analysis described in Sec. II C 2 found for QMF theory can be extended to the PQMF case with the replacement of spectral properties of $A_{ij}$ by those of $B_{ij}$. For example, the epidemic threshold is given by

(20) $$\lambda_{\text{c}}^{\text{PQMF}}\Omega^{\left(1\right)}\left(\lambda_{\text{c}}^{\text{PQMF}}\right)=1,$$

where $\Omega^{\left(1\right)}$ is the largest eigenvalue of $B_{ij}$. One can check that this result is exactly the same presented in Ref. [26] expressed in a different way. For $\lambda ≳\lambda_{\text{c}}^{\text{PQMF}}$ we have that $\rho_{i}∼w_{i}^{\left(1\right)}$, where $\left\{w_{i}^{\left(1\right)}\right\}$ is the PEV of $B_{ij}\left(\lambda_{\text{c}}^{\text{PQMF}}\right)$ and $\rho ≃b_{1}(\lambda \Omega^{\left(1\right)}\left(\lambda_{\text{c}}^{\text{PQMF}}\right)-1)$, where $b_{1}\left(N\right)$ has the same form as Eq. (13) replacing $v_{i}$ by $w_{i}$. Thus, the IPR of $\left\{w_{i}^{\left(1\right)}\right\}$, denoted by $Y_{4}\left[B_{ij}\right]$, allows us to quantify the localization in the PQMF theory.

## III. RESULTS

### A. Accuracy of theoretical estimates for the epidemic threshold

**FIG. 2.**
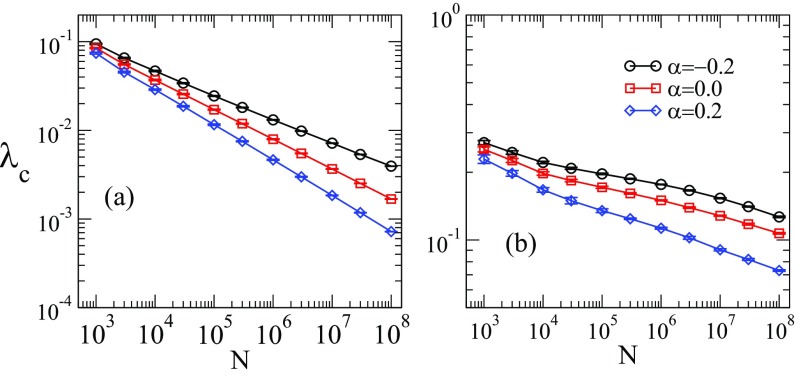
Threshold as a function of the network size for (a) γ=2.3 and (b) γ=3.5 and different values of α. The lower cutoff is $k_{\text{min}}=3$ for all curves, while the upper cutoff is $k_{\text{max}}=2\sqrt{N}$ for γ<3 and $k_{\text{max}}∼N^{1/\gamma }$ for γ>3. Curves are averages over ten networks; error bars are smaller than symbols.

Figure 2 shows the dependence of the epidemic threshold as a function of the network size obtained in simulations with different values of γ and α. We concentrate for the moment on two values of γ, representative of the cases γ<5/2 and γ>3, for which the physical mechanisms underlying the epidemic transition are clear [47]. Later we will discuss the case 5/2<γ<3, whose interpretation is hampered by extremely long crossover phenomena in the spectral properties. As we can see from this figure, all thresholds vanish as N diverges, regardless of the correlation level α and heterogeneity γ. Compared to the uncorrelated case, assortative networks (α>0) have a smaller threshold, while the threshold is larger for α<0, i.e., disassortative mixing, in agreement with the behavior of the LEV of the adjacency matrix [14,23]. In the case γ>3, this phenomenology can be qualitatively explained by considering the mechanism of long-range mutual reinfection of hubs [25,46,48], which triggers the epidemic transition. According to this mechanism, the subgraph consisting of the hub plus its nearest neighbors can sustain in isolation an active state for times long enough to permit the activation of other hubs, even if they are not directly connected. This mechanism is at work independently of degree correlations, as long as distances among hubs increase slowly enough with network size. In assortative networks, communication among hubs is enhanced since they have higher probability to be closer; for disassortative topology the converse is true and larger values of λ are needed to trigger the transition.

**FIG. 3.**
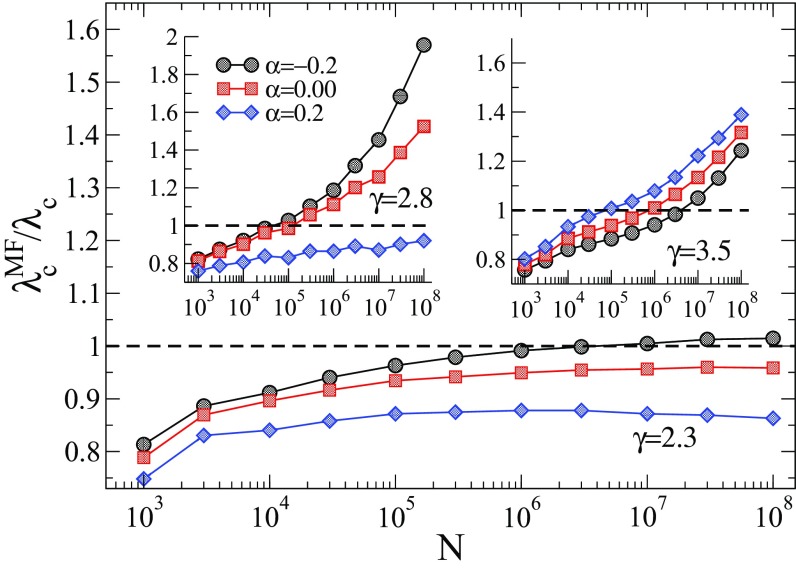
Ratio between thresholds of HMF theories ($\lambda_{c}^{\text{MF}}$) and simulations ($\lambda_{\text{c}}$) as a function of the network size for different values of γ and α. The main panel and the right and left insets correspond to γ=2.3, 2.8, and 3.5 respectively. An upper cutoff $k_{\text{max}}=2\sqrt{N}$ is considered for γ<3, while for $\gamma =3.5,\;k_{\text{max}}∼N^{1/\gamma }$. Averages correspond to ten network realizations and error bars are smaller than symbols.

The accuracy of HMF theory is tested with respect to simulations in Fig. 3. For γ=2.3, we see a non-negligible asymptotic discrepancy between HMF theory and simulations in the case of correlated networks. Interestingly, the HMF theory appears to overestimate the threshold for disassortative networks, while it underestimates it for assortative ones. For larger values of γ the discrepancy is conspicuous and the epidemic threshold is significantly overestimated, as can be seen in the insets of Fig. 3.

**FIG. 4.**
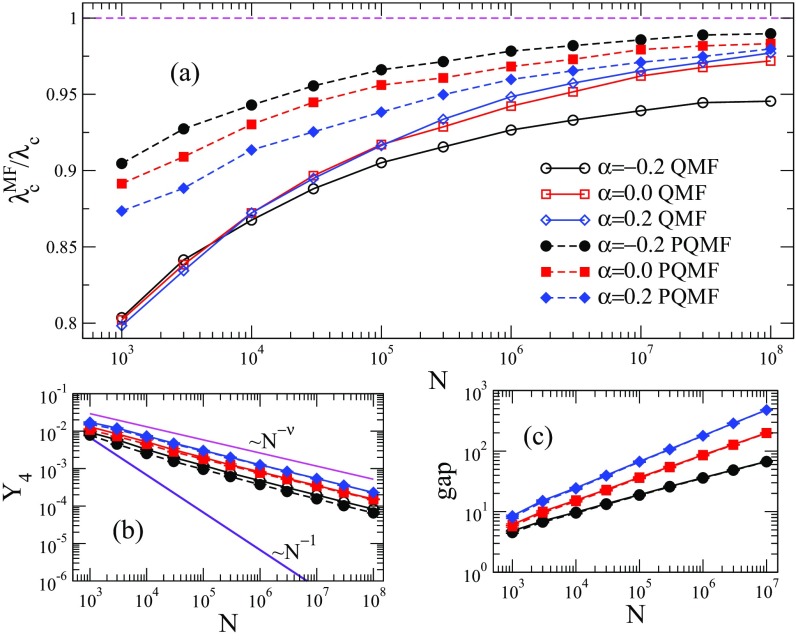
(a) Comparison of the QMF and PQMF mean-field theories, (b) IPR, and (c) spectral gap of $A_{ij}$ and $B_{ij}$ against size for γ=2.3 and different values of α. Averages correspond to ten network realizations. In (b), solid lines are power-law decays $Y_{4}∼N^{-\nu }$, with ν=(3−γ)/2 and $Y_{4}∼N^{-1}$ corresponding to localization in the maximum K-core and finite set of vertices, respectively. Solid lines and open symbols correspond to the QMF theory and $A_{ij}$ analysis, while dashed lines and closed symbols correspond to the PQMF theory and critical $B_{ij}$.

**FIG. 5.**
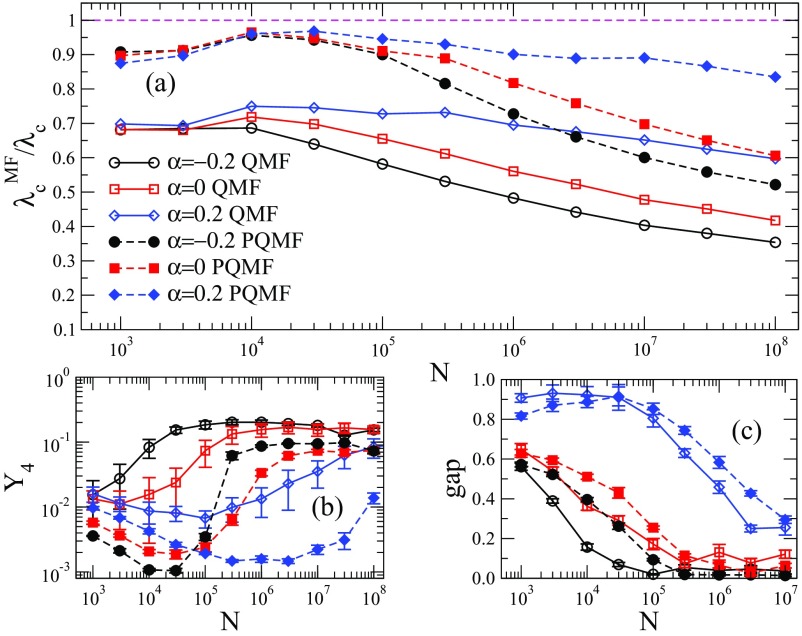
(a) Comparison of the QMF and PQMF theories, (b) IPR, and (c) spectral gap of $A_{ij}$ and $B_{ij}$ against size for γ=3.5 and different values of α, using an upper cutoff $k_{\text{max}}∼N^{1/\gamma }$. Averages correspond to ten network realizations. Solid lines and open symbols correspond to the QMF theory and $A_{ij}$ analysis, while dashed lines and closed symbols correspond to the PQMF theory and critical $B_{ij}$.

Comparisons between QMF and PQMF theories and simulations are shown in Figs. 4(a) and 5(a) in the range of network size $10^{3}\leq N\leq 10^{8}$. For γ=2.3, both QMF and PQMF theories appear to converge asymptotically to the epidemic threshold observed in simulations. The PQMF theory displays a faster convergence than the QMF theory, this effect being enhanced for smaller values of α. For γ=3.5, the predictions of PQMF and QMF theories succeed, qualitatively, in predicting that the threshold approaches zero in the thermodynamic limit even in the presence of correlations. However, the theoretical threshold estimates depart from simulation results leading to decreasing ratios $\lambda_{c}^{\text{MF}}/\lambda_{c}$ in the large network limit. We expect this ratio to decrease asymptotically as $1/\ln(k_{\text{max}})$ [46], in agreement with recent rigorous results [49]. Again, PQMF theory performs better than QMF theory. In this case, the improvement of PQMF over QMF theory grows with α.

### B. Relation to spectral properties

What is the origin of the discrepancies between theoretical predictions and numerical results observed in Sec. III A? In this section we investigate which spectral feature is correlated with the performance of the theoretical approaches. We consider both QMF and PQMF theories, testing their accuracy against the spectral properties of adjacency matrices $A_{ij}$ and $B_{ij}$, respectively.

Let us consider first the case γ=3.5. The real threshold is not the QMF one because the PEV is localized. As pointed out in Ref. [23], this in principle implies that the actual threshold coincides with the inverse of the largest eigenvalue corresponding to a delocalized PEV, coinciding with the HMF threshold $\lambda_{c}^{\text{HMF}}=\langle k\rangle /\langle k^{2}\rangle$. Actually, however, the QMF approach neglects dynamical correlations, which have the effect of allowing mutual reinfection events among different hubs in the network. In this way an endemic global state can be established due to the long-range interactions among localized states [46] setting the actual threshold to an intermediate value: $\lambda_{c}^{\text{QMF}}<\lambda_{c}<\lambda_{c}^{\text{HMF}}$. With this picture in mind, we can predict that, if the localization is stronger (higher values of the IPR $Y_{4}$), the actual threshold will be farther from $\lambda_{c}^{\text{QMF}}$ and thus the performance (accuracy) of the QMF approach will be reduced.

We plot the dependence of $Y_{4}$ on the system size N for γ=3.5 in Fig. 5(b): The IPR of $A_{ij}$ converges to a finite value in the thermodynamic limit, irrespective of the correlation degree, representing a PEV localized on a finite set of vertices [23,43]. The saturation with size occurs earlier for disassortative correlations and later for assortative, compared to the uncorrelated case. In general, for a given size $N,\;Y_{4}$ is larger for smaller α. As expected, a better QMF performance occurs for smaller $Y_{4}$.

The IPR analysis for the PQMF theory, involving $B_{ij}$, has a qualitatively similar behavior to QMF theory, but presents lower values for the IPR. Hence, the PQMF steady-state solution is less localized than that of the QMF theory. Correspondingly, the PQMF performance is better than the QMF performance. We also calculate, in Fig. 5(c), the dependence of the spectral gap on the system size, for both the adjacency matrix $A_{ij}$ (involved in QMF theory) and $B_{ij}$ (in PQMF theory). The spectral gap is defined as the difference $\Lambda^{\left(1\right)}-\Lambda^{\left(2\right)}$ between the largest and second largest positive eigenvalues of the adjacency matrices. The gap of the adjacency matrix $A_{ij}$ is small and it decreases as N grows, as predicted by Ref. [50]. The gap is smaller for smaller α. The dependence of the spectral gap of $B_{ij}$ on size is qualitatively similar to the gap of $A_{ij}$.

Notice that, while the amplitude of the spectral gap matters for the validity of the QMF prediction for the prevalence above the critical point [Eq. (12)], it does not play any role in the determination of $\lambda_{\text{c}}^{\text{QMF}}$. Therefore, there is no conceptual reason for expecting a correlation between QMF performance and spectral gap size. We find numerically such a correlation in Fig. 5, but we cannot attribute a causal meaning to it.

Let us consider now γ=2.3. In this case the physical mechanism underlying the epidemic transition is different, as it does not involve the interaction between distant hubs but rather the extension of activity from the maximum K-core to the rest of the network. The connection between QMF theory performance and localization is not easily predictable.

As shown in Fig. 4(b), the IPR for γ=2.3 follows a power law $Y_{4}∼N^{-\nu }$, with ν≈(3−γ)/2, which corresponds to the IPR localized in the maximum K-core of the network [43]. Correlations leave the scaling exponent unchanged, altering only the prefactor: The smaller the α, the smaller the IPR. This means that the PEV is still localized on a subextensive fraction of nodes. However, since $Y_{4}$ increases with α, the PEV is more localized for positive α than for negative. The same is true for the matrix $B_{ij}$ of the PQMF theory. Interestingly, the effect on the performance of the theoretical approaches is opposite. The QMF theory works better for larger $Y_{4}$ and the PQMF theory works better for smaller $Y_{4}$. We have no simple interpretation for this result.

Figure 4(c) shows the spectral gap for the WPCM networks with γ=2.3. In this case the gap increases with network size and it is smaller for smaller α. This is true also for the spectral gap of the PQMF theory. Finally, let us observe that there is almost no difference between the spectral properties of $A_{ij}$ and $B_{ij}$ for γ=2.3. This is indeed not surprising for α=0 since the term $\lambda^{2}k_{i}$ in the denominator of Eq. (19) is asymptotically negligible, because $\lambda_{c}^{2}k_{\text{max}}∼k_{\text{max}}^{2\gamma -5}\to 0$ as N→∞ for γ<5/2.

### C. Intermediate case 2.5<γ<3

**FIG. 6.**
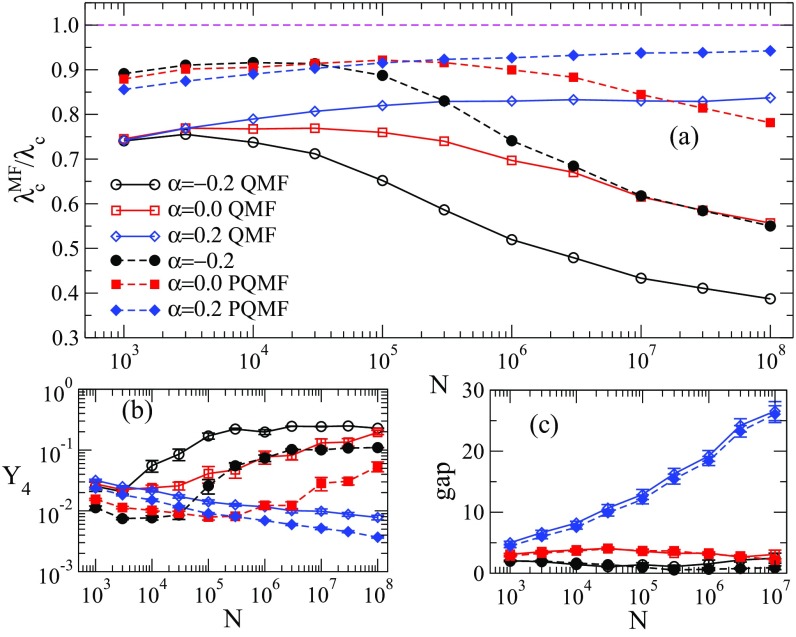
(a) Comparison of the QMF and PQMF theories, (b) IPR, and (c) spectral gap of $A_{ij}$ and $B_{ij}$ against size for γ=2.8 and different values of α, using an upper cutoff $k_{\text{max}}=2\sqrt{N}$. Averages correspond to ten network realizations. Solid lines and open symbols correspond to the QMF theory and $A_{ij}$ analysis, while dashed lines and closed symbols correspond to the PQMF theory and critical $B_{ij}$.

As for the other values of γ, in this range the vanishing of the threshold with N is observed regardless of the correlation pattern. The localization phenomenon of the PEV in the case 5/2<γ<3 is asymptotically analogous to the case γ>3. However, very strong crossover effects are observed in this case, because of the presence of a localization process on the maximum K-core (as for γ<5/2) competing with the localization around the hub [43]. As a consequence, already in the uncorrelated case, the PEV gets strongly localized around the largest hub only for very large values of N. Correlations further complicate the picture: Figure 6(b) shows that disassortative correlations accelerate the convergence to the final localized state. For α>0 instead, $Y_{4}$ is a decreasing function of N. The upward bend of the curve hints at an incipient crossover, but one cannot exclude that the asymptotic behavior is different for α>0. A similar pattern is observed for the case of the spectral gap [Fig. 6(c)].

With regard to the performance of the theoretical approaches, for negative or zero correlations the scenario perfectly matches what happens for γ>3: All theories somehow fail in capturing the way the threshold vanishes, with the PQMF theory being less inaccurate than the others. In the case α=0.2 numerical results seem to suggest that both theories describe quite well how the threshold changes with the system size. However, the large crossover effects mentioned above do not allow one to draw any firm conclusion.

We can summarize our findings by stating that the performance in predicting the behavior of epidemic threshold of the QMF and PQMF theories on WPCM networks is correlated with the size of the spectral gap and the IPR of the PEV of the respective $A_{ij}$ and $B_{ij}$ matrices that rule the prevalence near the transition point. A large spectral gap or a low IPR leads to a good performance of the mean-field theories, while the converse, small gap or large IPR, leads to deviations from the theoretical predictions. The QMF theory seems to be more correlated with the spectral gap, while the PQMF theory is with the IPR, at least in the regime where the gap is significant and the theories are accurate.

### D. Real networks

**FIG. 7.**
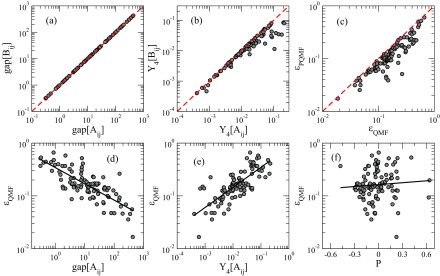
Scatter plots for a set of 99 real networks (see Appendix). Each point corresponds to a single network. (a) Spectral gap and (b) IPR of the matrix $B_{ij}$ plotted versus the corresponding values for the matrix $A_{ij}$. (c) Relative errors of QMF and PQMF theoretical predictions with respect to the simulation, defined by Eq. (21). Dashed red lines denote the diagonal. Relative errors of the QMF theory are plotted vs (d) the spectral gap, (e) the IPR, and (f) the Pearson coefficient.

We extend our analysis to a set of 99 real-world networks encompassing a broad range of origins, sizes, and topological features (see the Appendix). The spectral gap and IPR of matrices $A_{ij}$ and $B_{ij}$ are compared in the scatter plots shown in Figs. 7(a) and 7(b). We see that the spectral gap is almost the same for both adjacency matrices, while the IPR extracted from $B_{ij}$ is smaller than the one extracted from $A_{ij}$, in particular in the range of large IPR values. This shows that the PQMF matrices $B_{ij}$ are less localized than the matrix $A_{ij}$, relevant for QMF theory. The relative errors between the QMF or PQMF theory and simulations, defined as

(21) $$ɛ=\frac{\lambda_{c}-\lambda_{c}^{\text{MF}}}{\lambda_{c}},$$

are compared in the scatter plot shown in Fig. 7(c). As in the case of random networks, the PQMF theory outperforms the QMF theory for all networks investigated.

On this set of networks we test the relation observed for synthetic correlated networks, connecting qualitatively the accuracy of QMF and PQMF threshold predictions with the properties of the adjacency matrices (spectral gap and IPR), respectively, and with the Pearson coefficient P, measuring network topological correlations. Here P is defined as [2]

(22) $$P=\frac{\sum_{ij}\left(A_{ij}-\frac{k_{i}k_{j}}{N\langle k\rangle }\right)k_{i}k_{j}}{\sum_{ij}\left(k_{i}\delta_{ij}-\frac{k_{i}k_{j}}{N\langle k\rangle }\right)k_{i}k_{j}}.$$

The Pearson coefficient lies in the interval −1<P<1, being negative for disassortative, null for uncorrelated, and positive for assortative networks. The analyses for QMF theory are shown in the scatter plots of the relative error ɛ against the corresponding topological properties in Figs. 7(d) and 7(e). Qualitatively similar patterns obtained for PQMF theory are not shown. We can see that in real networks, the correlation between the performance of the theoretical prediction and the spectral gap is on average the same as that observed for the WPCM: A larger spectral gap is associated with a higher accuracy. The inverse correlation with the IPR is again preserved: A smaller $Y_{4}$ corresponds to a more accurate prediction. We do not find a significant correlation with the Pearson coefficient. Statistical analyses were performed using the correlation coefficients obtained from either power law, in the case of the spectral gap and IPR, or from exponential, in the case of the Pearson coefficient, regressions of the scatter plots. We obtain strong statistical correlations with |r|≳0.70 ($p<10^{-5}$) for both QMF and PQMF theories using either the IPR or spectral gap of the corresponding matrices. Values r≲0.2 (p>0.05) for correlation with the Pearson coefficient of the network confirm no significant statistical correlations.

### E. Epidemic prevalence near the epidemic threshold

**FIG. 8.**
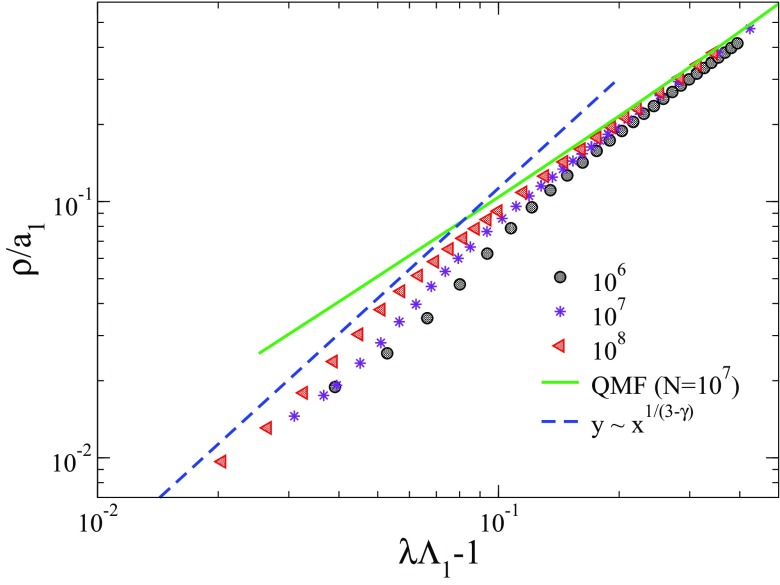
Rescaled average density as a function of the distance from the epidemic threshold. Quasistationary simulations for different sizes are indicated in the legend. The solid line is a numerical integration of the QMF theory [Eq. (9)] for $N=10^{7}$, while the dashed line is a power law with exponent predicted analytically in Ref. [51]. We used uncorrelated networks (α=0) with degree exponent γ=2.3 and $k_{\text{max}}=2\sqrt{N}$.

Figure 2 shows that the QMF prediction for the epidemic threshold tends to the same limit of numerical simulations for both uncorrelated and correlated networks for γ=2.3. This observation naturally leads to one wonder whether the QMF theory is asymptotically an exact description for SIS dynamics on random networks with γ<5/2. In order to answer this question we test the exactness of the other prediction of the QMF theory, Eq. (12), stating that the fraction of infected individuals decays to zero linearly as the threshold is approached from above. Numerical results, for the case of uncorrelated networks α=0 are shown in Fig. 8, where the density and the infection rates are rescaled to conform to Eq. (12). We can clearly see the existence of two scaling regimes. For $\lambda \Lambda_{1}-1\ll 1$ the density scales with an exponent larger than the prediction $\beta^{\text{(QMF)}}=1$. The observed exponent is consistent with the exact result of Ref. [51], β=1/(3−γ), which is also (probably accidentally) the value predicted by HMF theory [52]. This exponent is observed in a regime very close to the transition, where the system is kept asymptotically active only by virtue of the quasistationary method. We performed a nonperturbative analysis by integrating the QMF equations using a fourth-order Runge-Kutta method for $\lambda >\frac{1}{\Lambda^{\left(1\right)}}$ for $N=10^{7}$. A comparison with simulation results confirms that the QMF theory correctly predicts the linear behavior of the prevalence ρ around the epidemic transition, but only sufficiently far from it. In the immediate neighborhood of the threshold the decay is more rapid.

## IV. CONCLUSION

The determination of the epidemic threshold in models of disease spreading in complex topologies is a nontrivial problem in network science. Several theoretical approaches have been proposed, applying approximations with different levels of stringency, that provide contrasting predictions on the epidemic threshold. Among the main theoretical approaches at the mean-field level we can consider, in decreasing order of approximation, the heterogeneous mean-field theory, neglecting dynamical correlations and the actual pattern of connections in the network (preserving only its statistical properties); the quenched mean-field theory, also neglecting dynamical correlations but keeping the network structure; and the pair quenched mean-field theory, which incorporates dynamical correlations between pairs of connected nodes. In this paper we have presented a comparison of the predictions of these three approximate theories for the case of the susceptible-infected-susceptible epidemic model, focusing on the case of networks with a power-law degree distribution and degree correlations, representative of many real network systems.

Comparing the predictions with actual stochastic simulations of the SIS process, we observed that, independently of the degree of correlations, the predictions of PQMF theory are more accurate than those of QMF theory, while both outperform HMF theory, which fails to predict the vanishing threshold observed for a degree exponent γ>3. While overall PQMF theory is more accurate than QMF theory, the two approximations show different levels of accuracy when compared in networks with different levels of correlations. Thus, for the case of synthetic networks generated with the Weber-Porto algorithm [35], we observed that, for fixed network size and degree heterogeneity, QMF predictions are more accurate in assortative networks than in disassortative ones. On the other hand, PQMF theory is increasingly accurate in the presence of disassortative correlations for small degree exponent, while it is more accurate when correlations are assortative if the degree exponent is large.

We proposed a criterion for the accuracy of the QMF and PQMF approaches based on the spectral properties of the networks. We observed that the accuracy is positively correlated with the amplitude of the spectral gap of the adjacency matrix and is inversely related to the degree of localization of the principal eigenvalue, as measured by the inverse participation ratio. This general observation was corroborated by the analysis of a large set of real correlated networks, covering a wide range of sizes and topological features.

Additionally, we investigated the behavior of the order parameter of the transition, measured in terms of the prevalence or density of infected nodes in the steady state, for γ<5/2. We observe that, in uncorrelated synthetic networks, the linear decay (critical exponent β=1) predicted by QMF theory is observed in stochastic simulations not very close to the transition. When fluctuation effects become more important, i.e., very close to the transition, the observed exponent β crosses over to the value β=1/(3−γ), in agreement with rigorous mathematical results [51].

## APPENDIX: SUMMARY OF REAL NETWORK PROPERTIES

We consider 99 real networks with diverse structural properties, based on the lists of Refs. [53,54]. Here we investigate their giant connected components, after symmetrizing all edges (weighted and/or directed) and avoiding multiple and self-connections. The list of networks with some topological properties and epidemic (SIS) parameters is shown in Table I. For detailed information about the original references for all the networks, see Refs. [53,54].

**TABLE I.** Properties of the set with 99 networks of distinct types. We show the network size N, the average degree 〈k〉, the Pearson coefficient P, the IPRs of both $A_{ij}$ (${\mathrm{IPR}}_{\text{A}}$) and critical $B_{ij}$ (${\mathrm{IPR}}_{\text{B}}$) matrices, the spectral gap of $A_{ij}$ ($\Delta \Lambda_{\text{A}}^{1,2}$), and the thresholds of simulations ($\lambda_{c}$), QMF theory ($\lambda_{c}^{\text{QMF}}$), and PQMF theory ($\lambda_{c}^{\text{PQMF}}$).

Network	N	〈k〉	P	${\mathrm{IPR}}_{\text{A}}$	${\mathrm{IPR}}_{\text{B}}$	$\Delta \Lambda_{\text{A}}^{1,2}$	$\lambda_{c}$	$\lambda_{c}^{\text{QMF}}$	$\lambda_{c}^{\text{PQMF}}$
Karate club	34	4.59	−0.476	0.0730	0.0649	1.75	0.235	0.149	0.181
Radoslaw Email	167	38.9	−0.295	0.0133	0.0132	45.2	0.0191	0.0165	0.0168
Spanish B	12643	8.70	−0.290	0.0246	0.0174	47.5	0.0105	0.00897	0.00951
Spanish A	11558	7.45	−0.282	0.0190	0.0150	57.5	0.0113	0.00985	0.0103
US Air Transportation	500	11.9	−0.268	0.0176	0.0173	29.3	0.0251	0.0208	0.0214
Little Rock Lake	183	26.6	−0.266	0.0148	0.0145	14.6	0.0291	0.0242	0.0249
Japanese	2698	5.93	−0.259	0.0296	0.0214	21.7	0.0281	0.0233	0.0250
English	7377	12.0	−0.237	0.0120	0.0103	65.3	0.0101	0.00914	0.0094
French	8308	5.74	−0.233	0.0351	0.0200	26.3	0.0197	0.0165	0.0179
Jung	6120	16.4	−0.233	0.0478	0.0335	46.9	0.00810	0.00703	0.00743
JDK	6434	16.7	−0.223	0.0484	0.0341	47.6	0.00810	0.00696	0.00737
Political blogs	1222	27.4	−0.221	0.00701	0.00687	14.1	0.0153	0.0135	0.0137
Internet	22963	4.22	−0.198	0.0146	0.0116	18.4	0.0165	0.0140	0.0148
AS Caida	26475	4.03	−0.195	0.0240	0.0140	18.5	0.0173	0.0144	0.0157
EU email	224832	3.02	−0.189	0.00340	0.00328	15.2	0.0107	0.00975	0.0101
UC Irvine	1893	14.6	−0.188	0.00643	0.00608	28.6	0.0233	0.0208	0.0214
Linux, mailing list	24567	12.9	−0.185	0.00395	0.00386	147	0.00490	0.00448	0.00452
AS Oregon	6474	3.88	−0.182	0.0868	0.0429	19.1	0.0281	0.0216	0.0249
Linux, soft.	30817	13.8	−0.175	0.0256	0.0197	94.1	0.00670	0.00585	0.00616
Gnutella, 25 August 2002	22663	4.83	−0.173	0.000815	0.000464	1.79	0.108	0.0916	0.104
Les Miserables	77	6.60	−0.165	0.0492	0.0482	3.05	0.123	0.0833	0.0919
Petster-cats	148826	73.2	−0.164	0.00687	0.00635	405	0.000900	0.000847	0.000855
*C. Elegans*, neural	297	14.5	−0.163	0.0189	0.0176	10.1	0.0511	0.0410	0.0434
Libimseti	220970	156	−0.139	0.000406	0.000398	348	0.00110	0.00106	0.00106
David Copperfield	112	7.59	−0.129	0.0473	0.0397	7.57	0.103	0.0760	0.0844
Political books	105	8.40	−0.128	0.0444	0.0419	0.313	0.133	0.0838	0.0927
Google	15763	18.9	−0.122	0.0430	0.0303	65.1	0.00670	0.00575	0.00608
Social 3	32	5.00	−0.119	0.0665	0.0568	2.16	0.265	0.167	0.205
Euron	33696	10.7	−0.116	0.00379	0.00361	43.9	0.00910	0.00844	0.00859
Web Stanford	255265	15.2	−0.116	0.0245	0.0230	117	0.00250	0.00223	0.0023
Bay Wet	128	32.4	−0.112	0.0151	0.0147	25.6	0.0301	0.0252	0.0259
Bay Dry	128	32.9	−0.104	0.0148	0.0145	25.7	0.0301	0.0249	0.0256
Gnutella, 30 August 2002	36646	4.82	−0.104	0.000672	0.000604	2.19	0.0897	0.0773	0.0856
Gnutella, 31 August 2002	62561	4.73	−0.0927	0.000921	0.000731	1.85	0.0881	0.0759	0.0844
Petster-hamster	1788	14.0	−0.0889	0.0100	0.00938	21.6	0.0249	0.0217	0.0223
Petster-dogs	426485	40.1	−0.0884	0.00176	0.00157	300	0.00140	0.00135	0.00136
Network Science	379	4.82	−0.0817	0.0794	0.0705	2.21	0.230	0.0964	0.110
AS Skitter	1694616	13.1	−0.0814	0.00746	0.00709	251	0.00160	0.00149	0.00151
Slashdot zoo	79116	11.8	−0.0746	0.00229	0.00218	42.8	0.00830	0.00767	0.00778
Wikipedia, edits	113123	35.8	−0.0651	0.00295	0.00266	169	0.0027	0.00253	0.00255
CiteSeer	365154	9.43	−0.0632	0.0177	0.0110	4.54	0.0202	0.0172	0.0183
Cora	23166	7.70	−0.0553	0.0100	0.00898	3.66	0.0381	0.0317	0.0334
Thesaurus	23132	25.7	−0.0477	0.0017	0.00156	44.7	0.0105	0.0100	0.0102
DBLP, citations	12495	7.93	−0.0461	0.0282	0.0174	12.0	0.0277	0.0234	0.0251
Dolphins	62	5.13	−0.0436	0.0526	0.0493	1.26	0.231	0.139	0.164
DBpedia	3915921	6.42	−0.0427	0.201	0.0840	153	0.00190	0.00140	0.00180
Wikipedia, pages	2070367	40.9	−0.0418	0.00477	0.00294	194	0.00130	0.00124	0.00127
Epinions	75877	10.7	−0.0406	0.00219	0.00211	79.8	0.00570	0.00543	0.00548
Slashdot	51083	4.56	−0.0347	0.144	0.0347	20.2	0.0219	0.0170	0.0201
Hep-Th, citations	27400	25.7	−0.0305	0.00931	0.00705	20.3	0.00990	0.00899	0.00922
S 838	512	3.20	−0.0300	0.179	0.0340	0.889	0.382	0.200	0.297
Gowalla	196591	9.67	−0.0293	0.0180	0.00764	60.0	0.00650	0.00585	0.00609
Amazon, 12 March 2003	400727	11.7	−0.0203	0.118	0.0381	7.97	0.0273	0.0178	0.0227
Amazon, 6 June 2003	403364	12.1	−0.0176	0.0891	0.0279	7.87	0.0252	0.0175	0.0219
Amazon, 5 May 2003	410236	11.9	−0.0169	0.0843	0.0309	7.79	0.0249	0.0172	0.0214
Air traffic	1226	3.93	−0.0152	0.0191	0.0154	1.38	0.152	0.109	0.127
Gnutella, 4 August 2002	10876	7.35	−0.0132	0.00469	0.00377	4.26	0.0685	0.0586	0.0637
Gnutella, 24 August 2002	26498	4.93	−0.00778	0.214	0.0800	8.68	0.0865	0.0511	0.0700
Hep-Ph, citations	34401	24.5	−0.00644	0.00421	0.00323	3.61	0.0143	0.0131	0.0133
S 420	252	3.17	−0.00591	0.0542	0.0172	0.398	0.400	0.229	0.320
Amazon, 2 May 2003	262111	6.87	−0.00248	0.106	0.0114	0.771	0.0605	0.0425	0.0508
S 208	122	3.10	−0.00201	0.0419	0.0301	0.475	0.437	0.244	0.338
Digg	29652	5.72	0.00265	0.00457	0.00346	12.5	0.0369	0.0324	0.0348
US Power grid	4941	2.67	0.00346	0.0409	0.0386	0.874	0.396	0.134	0.157
Gnutella, 5 August 2002	8842	7.20	0.0146	0.00931	0.00855	7.68	0.0505	0.0425	0.0453
Jazz	198	27.7	0.0202	0.0143	0.0141	12.6	0.0301	0.0250	0.0257
Gnutella, 9 August 2002	8104	6.42	0.0331	0.00782	0.00737	13.1	0.0409	0.0351	0.0370
Gnutella, 8 August 2002	6299	6.60	0.0355	0.00795	0.00752	13.7	0.0413	0.0352	0.0371
LiveJournal	5189808	18.8	0.0394	0.00157	0.00157	42.7	0.00240	0.00186	0.00186
High school, 2012	180	24.7	0.0464	0.0102	0.0101	4.49	0.0401	0.0332	0.0344
Open flights	2905	10.8	0.0489	0.00963	0.00942	20.6	0.0181	0.0159	0.0162
Gnutella, 6 August 2002	8717	7.23	0.0516	0.0103	0.00957	3.20	0.0545	0.0447	0.0478
URV email	1133	9.62	0.0782	0.00956	0.00865	3.78	0.0581	0.0482	0.0512
High school, 2011	126	27.1	0.0829	0.0173	0.0171	11.6	0.0361	0.0294	0.0304
DBLP, collaborations	1137114	8.83	0.0964	0.00797	0.00840	0.0594	0.0113	0.00847	0.00855
MathSciNet	332689	4.93	0.103	0.0110	0.0103	1.56	0.0347	0.0277	0.0291
Social 1	67	4.24	0.103	0.0486	0.0418	0.975	0.292	0.179	0.223
Cond-Mat, 1993–2003	21363	8.55	0.125	0.0103	0.00947	7.41	0.0309	0.0264	0.0275
Protein 1	95	4.48	0.129	0.0723	0.0670	0.314	0.384	0.187	0.232
Cond-Mat, 1995–1999	13861	6.44	0.157	0.0163	0.0146	3.34	0.0509	0.0400	0.0424
College football	115	10.7	0.162	0.00977	0.00967	1.50	0.124	0.0928	0.102
Cond-Mat, 1995–2003	27519	8.44	0.166	0.00917	0.00847	6.09	0.0293	0.0248	0.0258
US Patents	3764117	8.77	0.168	0.0103	0.0100	8.05	0.0113	0.00885	0.00899
Facebook links	63392	25.8	0.177	0.00143	0.00140	25.8	0.00810	0.00754	0.00762
Cond-Mat, 1995–2005	36458	9.42	0.177	0.00814	0.00761	12.7	0.0223	0.0195	0.0201
Hep-Th, 1995-1999	5835	4.74	0.185	0.0523	0.0523	3.70	0.0913	0.0554	0.0587
AstroPhys, 1993–2003	17903	22.0	0.201	0.00447	0.00432	18.9	0.0117	0.0106	0.0108
Protein 2	53	4.64	0.209	0.0536	0.0500	0.722	0.305	0.172	0.210
Facebook wall	43953	8.30	0.216	0.00229	0.00214	7.86	0.0277	0.0252	0.0261
Dublin	410	13.5	0.226	0.0263	0.0261	3.62	0.0601	0.0428	0.0448
Actor coll. net.	374511	80.2	0.226	0.000600	0.000599	429	0.00120	0.00118	0.00118
Astrophysics	14845	16.1	0.228	0.00504	0.00494	5.64	0.0155	0.0135	0.0138
PGP	10680	4.55	0.238	0.0166	0.0163	4.25	0.0301	0.0236	0.0243
Hep-Th, 1993–2003	8638	5.74	0.239	0.0312	0.0312	8.03	0.0669	0.0322	0.0333
Reactome	5973	48.8	0.241	0.00414	0.00413	27.2	0.00550	0.00481	0.00483
Flickr	105722	43.8	0.247	0.00105	0.00105	101	0.00170	0.00162	0.00163
*E. coli*, transcription	97	4.37	0.412	0.0854	0.0807	0.327	0.328	0.153	0.184
Hep-Ph, 1993–2003	11204	21.0	0.630	0.00389	0.00389	153	0.00450	0.00408	0.0041
GR-QC, 1993–2003	4158	6.46	0.639	0.0209	0.0209	7.49	0.0273	0.0219	0.0225
